# Lectures on the Recent Advances of Our Knowledge in the Diagnosis and Treatment of Functional Nervous Affections

**Published:** 1866-05

**Authors:** C. E. Brown-Sequard


### LECTURES ON THE RECENT ADVANCES OF OUR KNOWLEDGE IN THE DIAGNOSIS AND TREATMENT OF FUNCTIONAL NERVOUS AFFECTIONS.

By C. E. BROWN-SEQUARD, M.D., F.R.S., &c.

---

### GENERAL REMARKS ON THE CAUSES, DIAGNOSIS, AND TREATMENT OF FUNCTIONAL NERVOUS AFFECTIONS.

GENTLEMEN:—The principal objects of these lectures are to point out the practical value of the great advances recently made in our knowledge of the normal and morbid states of the vital properties and functions of the various parts of the nervous system, and to show what progress we owe to experiments on animals, and to clinical researches on the therapeutic effects of many remedies, old and new. Before entering, however, into the study of our knowledge of the diagnosis and treatment of each type of the functional nervous affections, I propose to examine, in this lecture, some of the general principles of symptomatology, of diagnosis, and of treatment, in almost all the varieties of these affections.

One of the most important of the recent advances in physiology and pathology consists in the demonstration that the nervous conductors of the various kinds of sensitive impressions and of the reflex phenomena, and also for the transmission of nervous force to muscles, bloodvessels, etc., are absolutely distinct one from the others as regards their functions. Leaving aside the nerve-fibres of the brain, some of which have functions altogether different from those of other parts of the nervous system, I have ascertained that, besides the four distinct kinds of nerve-fibres of the higher senses, there are at least eleven distinct kinds of nerve-fibres in the spinal cord and in the cranial and other nerves. The following table shows what are the distinct functions of these eleven kinds of nerve-fibres.* They are—

1st. Conductors of impressions of touch.
2d. " " tickling.
3d. " " pain.
4th. " " temperature.
5th. " " contraction (muscular sense).
6th. Incito-motor conductors.
7th. Incito-nutritive and secretory conductors.
8th. Voluntary motor conductors.
9th. Involuntary motor conductors.
10th. Vaso-motor conductors.
11th. Nutritive and secretory conductors.

I hardly need to say that the number of distinct nerve-fibres is probably much greater than is shown by this table; but the demonstration of the distinction of other kinds of nerve-fibres (such as those serving to sensations of hunger, thirst, pressure, or to voluptuous sensations, etc.), is not yet sufficient.

Almost all the symptoms of functional (and I might say, also, of organic) nervous affections take place through one or other of three modes of alteration of the properties and functions of the fifteen kinds of conductors above named. Those three modes consist in: 1st, a diminution or loss of power; 2d, an

* I have shown that the nerve-fibres serving to sensations of touch, of tickling, of pain, and of temperature, are not only completely distinct one from the others, but that, after having entered the spinal cord as parts of the posterior roots of the spinal nerves, and after a complete decussation between the fibres of the two sides of the body in that nervous centre, they form in it distinct groups or columns of conductors; so that an alteration, limited to a small part of the spinal cord, may strike either the column for touch, or that for tickling, or one of the other two kinds of conductors of sensitive impressions. I have shown, also, that the conductors for the muscular sense differ from the conductors just spoken of in this respect, that they do not decussate in the spinal cord, but that they form also a distinct column in each lateral half of that organ. (See Journal de la Physiologie de l'Homme, etc., No. 24, vol. vi., pp. 610-19 and 645.)

increase of power; 3rd, a morbid state producing a great variety of phenomena. In the nine kinds of conductors serving for the transmission of sensitive impressions the first of these three modes of alteration constitutes *anæsthesia*, the second *hyperæsthesia*, and the third is the cause of *morbid sensations* (including the so-called *referred sensations*).

I will not enter into details here about the kinds of nerve-fibres which I call incito-motor, incito-nutritive or secretory, or about those which I simply name nutritive or secretory; but as it would be impossible to understand the future lectures of this course without some knowledge of the properties and functions of these nerves, I will give, as briefly as possible, a few notions concerning their physiological and pathological history.

1st. *Incito-motor nerves.*—These are the well-known excito-motor nerves of Marshall Hall. A great many facts prove that they are absolutely distinct from the sensitive nerves. All the reflex movements of the muscles of organic or animal life (including the excretory ducts of glands, the bloodvessels, etc.) originate through an irritation transmitted by these incito-motor nerves.

2dly. *Nutritive and secretory nerves, and incito-nutritive and secretory nerves.*—That more appropriate names should be given to these nerves, I will not deny; but so long as their real mode of action remains somewhat doubtful, I think it is better to call them by names which only imply what we positively know of them—*i. e.*, that they are agents of modification of secretion and nutrition. The *secretory and nutritive nerves* are in many respects the antagonists of the *vaso-motor nerves.* While these last nerves, when put in action, produce a contraction of bloodvessels and all the phenomena which I have shown to ensue from that contraction (such as a diminution in the quantity of blood, a diminution of sensibility and other vital properties and of secretions),* the nutritive and secretory nerves, on the contrary, when put in action, occasion a dilatation of bloodvessels and all the phenomena that ensue from it, as shown first by experiments of Czermak and of Cl. Bernard. I proposed long ago (in my lectures at the College of Surgeons in 1858)† an explanation of the action of these nerves, which becomes more and more probable with the frequent discovery of new facts concerning these nerves. The explanation is, that the secretory and nutritive nerve-fibres act upon the tissues so as to in-

† See my Course of Lectures above quoted, p. 149.

* See Course of Lectures on the Physiology and Pathology of the Central Nervous System, 1860, p. 142.

crease their chemical interchanges with the blood, and that, in consequence of this increase of chemical interchange, blood is more attracted to the part, and, as an effect of this greater afflux of blood, there is a dilatation of bloodvessels. In many of the future lectures I will mention phenomena due to this dilatation of bloodvessels from an irritation of this peculiar kind of nerves. I will only add now to what I have already said on this subject, that it is chiefly by a reflex action that these nerves produce their peculiar effects. The redness, the congestion of parts attacked with neuralgia or of neighboring parts, the secretion of tears when the eye is irritated or the skin of the face pinched, the secretion of mucous from the nose, or from laryngeal, tracheal or bronchial mucous membranes, or the diarrhœa produced by cold, and an immense number of facts in which there is a congestion or a secretion as a result of an irritation of cutaneous or other nerve fibres, are all effects of the peculiar influence exerted by secretory and nutritive nerve-fibres on glands and other tissues.

These nutritive and secretory nerve-fibres are, as I will try to prove in future lectures, the agents of production of inflammation, suppuration, and ulceration, by a reflex action. They are the channels of production of meningitis, of encephalitis or myelitis, when those inflammations are due to cold, to a burn, or to a visceral affection. They also have a great share in the production of many functional nervous affections, and particularly tetanus. Besides, through the agency of these nerves, much may be done for the treatment of nervous affections, as I will show hereafter.

The incito-secretory and nutritive nerve fibres are the incident or centripetal nerve fibres which, by a reflex influence, act upon the centrifugal secretory and nutritive nerve-fibres. Their distinct existence is not fully demonstrated, but many facts render it extremely probable.

The causes of functional nervous affections can be divided into several groups. 1st. An irritation by worms, by teething or decayed teeth, by cold, by a burn, a wound, an inflammation, a neuralgia, etc., of centripetal nerve-fibres (the incito-motor, the incito-nutritive, and some others). 2d. An alteration in the quantity or the quality of blood. 3d. Both the two preceding kinds of causes coëxisting together, as in cases of typhus fever, of variola, of diphtheria, of uræmia from disease of the kidneys, etc.

I will not now insist on the mode of action of these causes, as that will be better done in treating of each nervous affection separately; but there are a few general remarks which will be in their proper place in this introductory lecture. There are two general rules which particularly deserve attention:—

1st. *The same peripheric cause of irritation acting on the same centripetal nerve may produce the greatest variety of effects, including every functional nervous affection or disorder.* This is well illustrated by the effects on the eye of a neuralgia of the infraor supra-orbitalis nerves. There are cases showing: 1st, spasm of the sphincter of the pupil; 2d, mydriasis; 3d, spasm of the orbicularis palpebræ; 4th, paralysis of the orbicularis; 5th, paralysis or spasm of one or several of the muscles of the ocular globe; 6th, photophobia; 7th, amblyopia or amaurosis; 8th, congestion or inflammation of the conjunctiva or of other parts of the eye; 9th, diminution or increase of the secretions of the lachrymal and other glands; 10th, a cataract; 11th, a glaucoma.

Other instances, though less striking, might be adduced. I will only mention what we know as regards the effects of cold air on persons coming out of a theatre. One may be attacked with a sore throat, a second with opthalmia, a third with enteritis, a fourth with a nephritis, and many others with any other visceral inflammation; while one may be attacked with a facial paralysis, and others with almost every other partial paralysis, or with chorea, with contracture, with meningitis, or an inflammation of some part of the nervous centres. It is true that here it is not the same part of the skin which is acted upon by the sudden lowering of the temperature; in most of such cases, however, it is the front part of the chest and the neck that are subjected to the influence of the cold air.

It is probable that the great variety of phenomena following an irritation of centripetal nerve-fibres depends on differences of excitability of some parts of the nervous centres in different individuals. I have examined carefully, on a large number of men, the effects of tickling the sole of the foot, and found that these phenomena differed considerably on different individuals. In one, laughter predominated; in another, involuntary screaming, or shedding of tears, or jerks either in the irritated limb or in both lower limbs, or a general trembling, or a spasm of the diaphragm, or an almost tetanic rigidity of the irritated limb. I need not add that in some individuals there was hardly any effect produced when, being prepared for the irritation, they fought against its influence—a fact which, with many others, shows that by an effort of the will we sometimes can, at least in a certain measure, prevent the production of reflex phenomena.

I will not say more at present on this subject, leaving for other lectures the demonstration that it is through a real reflex action (*i.e.*, through a mechanism similar to that of reflex movements) that the morbid effects of an irritation of centripetal nerve-fibres take place.*

2d. *The degree of excitability of the different parts of the nervous system may increase or decrease considerably in the same person under the influence of various causes.* This proposition is of so great an importance in the diagnosis and treatment of nervous affections, that it is only by the light it throws on many otherwise obscure cases that we are enabled to recognise their nature, and to apply the proper treatment. Many parts of the nervous system that are completely, or almost completely, inexcitable in a healthy condition, become excitable, and sometimes in a wonderful degree, under the influence of several morbid causes. Amongst these parts, I will point out the grey matter of the spinal cord, and the nerves of the tendons, aponeuroses, dura mater, periosteum, bowels, bladder, kidneys, and some other viscera. Many causes very different one from the other, may increase the excitability of the various parts of the nervous system. I will mention here only the principal of these causes: 1st. I have found that muscles, nerves, and the spinal cord, become more excitable when laid bare, and particularly when the air in contact with them is richer in oxygen than the ordinary atmospheric air.† 2d. A congestion or an inflammation will increase the excitability of nervous tissues everywhere, but nowhere so markedly as in the grey matter and some parts of the white columns of the spinal cord, which in consequence of that increase, will become able to produce referred sensations of pain, of cold or heat, of tickling, etc., and phenomena due to an irritation of motor, vaso-motor, and nutritive nerves. 3d. An afflux of blood, such as occurs merely by gravitation, or after a section or a paralysis of the sympathetic nerve, or a lesion of the spinal cord, the medulla oblongata, or the base of the brain, will also increase the excitability of peripheric nerves in the parts where this affiux takes place. 4th. Certain remedies or poisons (strychnine particularly) will increase the reflex excitability of the spinal cord to a wonderful degree; while others, such as atropine, chloroform. etc., will diminish it consider-

* In one of the ensuing lectures I will explain why I do not admit the views so ably propounded by Prof. Lister and Dr. Handfield Jones on the so-called "inhibitory" nerves.

† See Proceedings of the Royal Society, vol. viii. 1857, p. 598; and Journa l de la Physiol. de l'Homme, &c., vol. i. 1858, p. 617.

ably. 5th. Certain diseases, such as tetanus and hydrophobia, will increase extremely the reflex excitability of some parts of the cerebro-spinal axis. 6th. A great loss of blood, anæmia, chlorosis, will also increace the reflex excitability of the nervous centres.

The increase of reflex excitability in cases of extreme debility, as in old age, or after a loss of blood, or other causes of insufficient nutrition, would be very difficult to understand if we did not know that the reflex excitability of the spinal cord can be increased under the influence of certain substances (strychnine principally) when no blood at all remains in the bloodvessels of that nervous centre, as proved beyond the possibility of doubt by Messrs. Martin-Magron and Buisson. The excitability of sensitive nerves may also be increased when the quantity of blood is much diminished, as we often observe in fingers that have been exposed to cold air or cold water. It seems, from a review of all the facts I know bearing on this point, that certain substances contained in blood, altered in quantity or quality, will act on the excitability of nerve-fibres in the nervous centres, or in the nervous trunks and branches, so as either to increase it, as is done by strychnine (on the grey matter,) and oxygen (everywhere), or to decrease it, as is done by carbonic acid, atropine, chloroform, etc.

To conclude what I wish now to say on the excitability of the nerves, I will only mention—1st, that I have ascertained that the excitability of the same nerve varies in different parts of its length, and to such a degree that in some parts the excitability seems nil or is very slight, while in other parts it is considerable; 2d, that I have shown by positive experiments that the excitability of muscles, of nerves, and of the spinal cord, may be very much increased, while the *force* developed by the action of those parts is very small. For instance, atrophied muscles, unable to contract with half the force shown by healthy muscles, will, however, contract under the influence of an excitation that will produce no effect on healthy muscles.

I will say only a few more words on the causes of functional nervous affections. The important discussions between Virchow, Spiess, and others, on the share of the nervous system in the causation of the various morbid alterations of tissues and organs, have been very useful in bringing forward many interesting facts; but the exclusiveness of the two opposite schools, at the head of which are the eminent men I have just named, has thrown a great deal of obscurity on questions which, considered with less partiality, might have been solved easily. It is certainly true, as maintained by Virchow,* that nutrition and secretion, normal and abnormal, can be carried on without the intervention of the nervous system; but this does not at all prove that the nervous system cannot interfere, for good or for evil, in the nutrition and secretion in the various tissues and organs. For instance, there is no doubt whatever that an inflammation, followed or not by suppuration and ulceration, can take place without any intervention of the nervous system: but, as will be proved in one of the ensuing lectures, there is no doubt also that inflammations not only can be, but very frequently are, produced by a nervous agency. Indeed, facts are extremely numerous that establish clearly—1st, that normal nutrition and secretion do not depend essentially on any interference by the nervous system, and that all morbid changes in these fundamental organic functions can take place without any nervous influence; 2d, that the nervous system may, and almost constantly does, influence nutrition and secretion, and that it frequently produces, or helps to produce, a great variety of alterations of these two fundamental functions.

Not only can an excitation of the same nerve produce effects in the different parts of the nervous centres, in one or other of the viscera, or in distant nerves, or muscles, or bones, etc., but it can also produce, in the same part of the nervous centres, in the same viscus, in the same muscle, etc., different kinds of alteration. Why is there such a variety of alterations produced in one and the same part by an excitation which varies only in intensity? To answer this question I must go beyond the limits of the subject-matter of this course of lectures, and cast a glance on the mode of production of morbid affections of the various tissues and organs. Physiology, morbid anatomy, and clinical observation clearly point out that all nervous affections (organic and functional), as well as affections of any other part of the body, owe their production to three different causes.

1st. A special inherent tendency (inherited or not) of the elementary parts of the tissues to become altered in one or in another way.

2d. The production, or introduction in the blood, of those materials that are necessary for the formation of morbid growths, or able, like poisons, to cause alterations of nutrition or secretion.

3d. An influence of an excitation from outside, acting with or without the intervention of the nervous system, or a purely

* See Dr. Chance's excellent translation of Virchow's "Cellular Pathology," Lect. XIV. London, 1860.

nervous influence starting from a peripheric or a central part of the nervous system.

If we keep in mind the truth that these three causes may exist together and in various degrees, we can easily understand how the same excitation of the same nerve may produce in the same distant part different kinds of alteration of nutrition. It is so that an irritation from a wounded nerve will produce either tetanus, or cholera, or catalepsy, or epilepsy, or delirium, etc., and by a reflex action on a peripheric part, muscular wasting or trembling, a contracture, a neuralgia, etc.

I now pass the diagnosis of functional nervous affections, on which subject I will only point out in parallel columns the principal distinctive features of these affections compared with organic nervous diseases.

| *Characters of functional nervous affections.* | *Characters of organic nervous diseases.* |
| --- | --- |
| 1st. The principal causes are: an alteration of the blood, and an irritation of a part of an incident or centripetal nerve, by a neuralgia, by worms, by teething or decayed teeth, by a wound, a burn, &c. | 1st. One of the principal causes is a special tendency (inherited or not) to inflammation, to alterations of the bloodvessels, or to the formation of morbid growths. |
| 2d. Great variability in the intensity of the symptoms, and regular or irregular recurrence of attacks, with intervals of almost perfect health between these attacks. | 2d. Persistence of the principal symptoms, with slow variations in their intensity. |
| 3d. A sudden or rapid cure or improvement is not rare. | 3d. A sudden or almost sudden cure is impossible, and a rapid improvement is exceedingly rare. |
| 4th. Certain symptoms—such as a sensation of pricking, of formication, of burning heat, of icy cold, and other symptoms of irritation of conductors of sensitive impressions, and also alterations of nutrition and secretion of the skin and of mucous mem- | 4th. Most of the symptoms due to the irritation of conductors of sensitive impressions and of nutritive and secretory conductors, are extremely frequent in inflammation and even congestion of the nervous centres or their meninges, and they are not |

| | |
|---|---|
| branes and glands (bladder, kidneys, etc.) are extremely rare, except in a few of these affections (neuralgia, affections due to alterations of the blood, etc.). | rare in many other organic nervous diseases. |
| 5th. The temperature of affected parts generally low. | 5th. The temperature of affected parts generally high. |
| 6th. The sphincters of the bladder and rectum usually normal. | 6th. The sphincters of the bladder and rectum often attacked with spasm or paralysis. |
| 7th. An aura, felt or unfelt, very frequently exists in some forms or periods of epilepsy, of hysteria, of catalepsy, of hydrophobia, etc. | 7th. An aura, felt or unfelt, exists only when the organic disease has caused a functional nervous affection. |
| 8th. To remove the cause is an essential part of the treatment. | 8th. To remove the cause is often impossible, and, when possible, of less importance than the direct treatment of the structural alterations. |

Other means of diagnosis between organic and functional nervous affections have been found within the last few years. As I shall have to speak of them at some length when I treat particularly of certain functional disorders, I will merely say here that the most interesting amongst them are—1st. The effects of pressure on nerves, as employed by Dr. A. C. Pinel, Dr. Aug. Waller, and Messrs. Bastien and Vulpian. 2d. The effects of tickling, and the degree and extent of reflex movements. 3d. The influence of galvanic applications. 4th. The effects of strychnine as a test for congestion in the spinal cord and its meninges. 5th. The existence of anæsthesia limited to a small spot. 6th. The alteration in the speed of transmission of sensitive impressions. 7th. The existence of an unfelt aura.

*Treatmemt of functional nervous affections.*—Great advances in the treatment of neuroses have been made within the last ten or fifteen years, particularly as regards a more rational or better appropriated employment of remedies and other modes of treatment than by the discovery of new remedies. I may say that I will speak of the rejection of certain modes of treatment, or of their limitation to fewer cases, as a real advance in therapeutics. I will divide this subject into eleven parts, as follows:—

I. Means of suppressing the causes, or of diminishing their intensity.

II. Means of diminishing the reflex excitability of the nervous centres.

III. Moral treatment.

IV. Special modes of treatment in periodical affections.

V. Treatment through irritations of the sensitive and other incident nerves.

VI. Physical and mechanical means of treatment.

VII. Complex modes of treatment, combining the two processes of irritation of incident nerves, and a modification of the blood.

VIII. Change of the composition of the blood, and elimination of morbid and other poisons.

IX. Special use of anæsthetics.

X. Treatment by remedies acting directly on the nervous system, or on the unstriped muscular fibres.

XI. Treatment by tonics and other remedies.

I. *Means of suppressing the causes, or of diminishing their intensity.*—When functional nervous affections are due to an evident irritation of a branch, or of the terminal ramifications of a nerve of a limb, or a superficial nerve of the abdomen or chest, several means of treatment may be successfully employed to check or to cut off the irritation. I will say a few words on the most important of these means.

1st. *Local applications of narcotics.*—In cases of epilepsy, of tetanus, of hysteria, and most other functional affections of the nervous system, a wound of the skin or of a branch of a nerve may be the cause of the nervous disorder. Narcotics, and particularly soluble salts of morphia and atropia, employed together, should be applied on the wound itself, in doses varied according to the absence or abundance of suppuration. An important rule of this mode of treatment, about which I will say much more in the lecture on tetanus, is that the application of narcotics must be frequently renewed, particularly if there is an abundant discharge of pus. If the cause of a functional nervous affection is a division of a large branch or of the trunk of a nerve, an injection of a solution of morphia and atropia should be made at some distance from the wound in the subcutaneous cellular tissue, round the central part of the divided nerve (half a grain of a salt of morphia with one-fiftieth of a grain of a salt of atropia).

2d. *Local application of ice.*—I have long ago pointed out the usefulness of this application on a wound producing a functional nervous affection. I will only say here, that when such a means is employed, particularly in a case of tetanus, there should be no interruption in the presence of ice on the wound during the whole of the time that the nervous affection lasts. Billroth has recorded two cases in which tetanus due to a wound has appeared, notwithstanding the application of ice on the wound. It is probable that in these cases there had been some interruption in the application of ice.

3d. *Application of the actual cautery.*—This means, which may be useful when it is necessary to alter the nature of the secretions in a wound, or to destroy parts of tissues containing a venom, has not generally so much value as the preceding and the following modes of treatment.

4th. *Various applications on the trunk of nerves at some distance from a wound.*—It may be useful to lay bare the nerve that gives filaments to the wounded part, and to apply sulphuric ether or narcotic alkaloids or ice upon it. In cases in which there is reason to expect that the original wound will soon heal, this mode of treatment might prove useful.

5th. *Section of a nerve.*—The number of cases of epilepsy, of tetanus, and of other nervous complaints, due to a wound, a burn, etc., in which this mode of treatment has proved quite successful, is now so large that there is no doubt as regards its immense value. It is most important to know that the operation must be performed early, and that its chances of success decrease rapidly with the prolongation of the nervous affection produced by a wound, a burn, or some other peripheric cause of irritation. It is necessary not only to divide the nerve completely, but to take away a small part of its peripheric end, which is to be examined carefully with the microscope to ascertain whether it is altered or not. If it is found inflamed, or otherwise altered, the operation must be repeated (if possible, of course) on the same nerve, much nearer the spine or the cranium. The examination of a small part of the nerve extirpated in this second operation will prove important for the prognosis of the case. It can hardly be an objection against the division of a nerve in affections like epilepsy, tetanus, hydrophobia, etc., that a paralysis is the necessary result of the operation. No sane man can hesitate between these two things—an almost certain death, or the persistence of a most horrible affection which may produce imbecility; and a paralysis of motion and sensation in a part of a limb, or even in a whole limb. And the hesitation, if any could exist, would certainly give way to the knowledge that the ends of a divided nerve, even when a small part has been excised, will often unite soon, or at least within a year, and the paralysis be cured more or less completely. The rapidity of union of the ends of a divided nerve may be so great that in a few weeks, and even sooner, there may be a partial return of function, as shown by cases reported by Mr. J. Paget, by Mr. Syme, and by Prof. Laugier. As regards the completeness of the return of the vital properties in a divided nerve, I have seen that it may be as perfect as possible, particularly in the case of a nobleman on whom Sir William Fergusson had divided the infra-orbitalis for a neuralgia.

6th. *Operations on the genital organs.*—An able surgeon has lately treated leveral kinds of functional nervous affections by the extirpation of the clitoris. That this operation may sometimes be useful, there is no doubt at all. But I cannot look upon this mode of treatment as one that should be employed in other cases than those in which a distinct aura starts from the clitoris, or in those cases in which that organ is morbidly sensitive and much hypertrophied. There are cases of nervous complaints, due to masturbation, in which the clitoris has been extirpated without any durable benefit as regards the nervous affection, or even as regards the masturbation. In women, as well as in men, the only decisive means against masturbation is the production of a small ulcer (by caustics or the red iron) on parts of the genital organs that are unavoidably touched or moved in the act of self-abuse, so that every attempt to accomplish the act, either with or without the help of the hand, is so painful that the patient must give it up. As regards the removal of the testicles, it seems to me a barbarous operation if performed only because there is an excessive tendency to sexual intercourse. I will say more on this point when I treat of epilepsy.

7th. *Trephining of the cranium.*—As a mode of treatment against epilepsy, this operation has been much more frequently performed than is generally known. It has also been performed, and successfully, in a case of tetanus, which I will mention in another lecture. It would be out of place here to discuss the question of the usefulness of that dangerous operation as a means of treatment of epilepsy. I will merely say that it is only in cases of an irritation of the dura mater, by a broken piece of bone, by diseased bone, or any other evident organic cause, that trephining can rationally be performed; and that even in such cases a cure might be obtained (and has really been sometimes obtained) by the use of counter-irritants on the diseased spot. In another lecture I will give the details of a most interesting case published by Mr. Henry Lee (in Dr. Beale's *Archives of Medicine*, 1860, p. 80), in which trephining of cranium cured an ulcerous affection of the skin of the arm, and spasmodic movements in the same limb.

8th. *Ligature of the carotid artery.*—This most irrational mode of treatment is, or, I hope, will be, completely abandoned. It has been employed in epilepsy, and in mania, with the view that those affections depend on a congestion of the brain, and that a ligature round the carotid artery would diminish that congestion. I will show in the lectures on Epilepsy that the good effects of this operation in the cases of Preston and his imitators have been obtained chiefly through some injury to the cervical sympathetic nerve.

9th. *Other operations for the removal of causes of functional nervous affections.*—I will only point out the importance of the removal of a decayed tooth, of a tumor, of a carious or necrosed bone in those cases in which there is a probability that these sources of irritation are the principal causes of a nervous complaint. Of other operations, such as tracheotomy and the cauterization of the urethra, I will only say a few words. It is now perfectly established that the theory of epilepsy given by Marshall Hall was wrong; and that if tracheotomy may be useful in some cases of epileptic coma, of spasm of the glottis, in tetanus, in hydrophobia, or in whooping-cough, etc., this operation is then of service against an effect, and not against a cause, of the existing nervous affection. As regards the cauterization of the urethra, according to Lallemand's plan, in cases of nervous complaints due to seminal losses, I must say that I have been consulted by a great many patients who had been vainly submitted to that operation; while I have often observed a considerable amelioration, and sometimes a cure, by a medicinal and hygienic treatment, consisting in the use of atropine, the ergot of rye, large doses of the bromide of potassium, and tonics—such as quinine, iron, manganese, silver, with cold, shower, and sitz baths, gymnastic exercise, and the most nourishing alimentation.

10th. *Treatment against alterations of the blood, and visceral diseases*—I only wish to point out under this head that every organ or part of an organ, in so far as it has nerves, and also if it has any marked influence on the composition of the blood, can be the cause of a functional nervous affection. Therefore any alteration of any organ must be fought against in any such nervous affections, particularly when no other cause of it can b found but that alteration. As regards the morbid states of the blood, there is no general rule of treatment that can be given. Anæmia, chlorosis, rheumatism, gout, and syphillis are to be treated in the same way when they are the causes of a nervous complaint as when they have produced no such effect.

II. *Means of diminishing the reflex excitability of the nervous centres.*—An increase of the reflex excitability of some part of the nervous centres, is one of the most important elements of many neuroses, and particularly epilepsy, hysteria, tetenus, hydrophobia, delirium tremens, chorea, paralysis agitans, and some forms of reflex insanity. To diminish that increase of reflex excitability is an essential part of the treatment in those affections. The following remedial agents are to be employed against this morbid excitability:—1st. Codeine, morphine, atropine, valerian aconite, the chloride of barium, and the bromide of potassium, are undoubtedly the most reliable remedies against an increased reflex excitability. According to the kind of nervous affection, and also to the seat of the increase of that vital property, we are to select one or several of these remedies. Atropine, valerian, and the bromide of potassium, are the most valuable in epilepsy; the chloride of barium is of real value against tetanus and paralysis agitans, but of no use in the common forms of epilepsy; codeine, morphine, and valerian are useful against hysteria, etc. None of these remedies, however, equals chloroform, but unfortunately its influence is merely transitory. Counter-irritants and the warm bath have also a great power against the increased reflex excitability, as I will show in another part of this lecture. 2d. As a morbidly increased excitability is very often due to anæmia, or to an impoverished nutrition, all the hygienic and medical means (good food, exercise, and tonics) that can improve nutrition, should be ordered in such cases against that morbid state. 3d. It is of the utmost importance to improve the sleep, which is generally so bad in patients attacked with a morbid increase of the reflex excitability. For this purpose an invaluable remedy has recently been discovered: it is the bromide of potassium. Excepting when pain is one of the causes preventing sleep (in which case opium, aconite, and belladonna should be employed together), I have found that this remedy has a most wonderful power to produce a quiet and refreshing sleep, without any drawback that I am aware of. I usually give to adults a dose of thirty grains of that salt a-quarter of an hour before the last meal, and a second dose of from thirty to fifty grains at bedtime. In cases in which, without any nervous complaint, there is sleeplessness owing to some cause of cerebral excitement, as well as in all neuroses, excepting hydrophobia, tetanus, very severe cases of delirium tremens, and some forms of insanity, sleep is almost always induced by that remedy. In some cases I have found it necessary to increase the dose of the bromide, and to give also one grain or one grain and a-half of codeine an hour before bedtime. In those affections in which the bromide of potassium is not powerful enough as a sleep-inducing agent, a warm bath of four, five, or six hours' duration is often successful.

III. *Moral treatment.*—As I shall have to dwell at length on this subject when I treat of hysteria, I will only say a few words here on two general principles, which, notwithstanding their importance, are very much neglected. The first of these principles, so well established by the researches of Dr. Cerise, is that a *serious aim* is of the greatest value, and for many persons quite essential, to prevent or to check nervous disturbances. The applications of this principle are, of course, very difficult, and often impossible, in certain neuroses; but in those cases in which any kind of serious work, not too fatiguing or exciting, is liked by the patient, he should be induced to do it. In cases of hypochondria, of hysteria, of chorea, and even of epilepsy, a great benefit can be derived from a serious employment of the mental and physical activity of the sufferer. How often have I not seen young epileptics kept in idleness (alas! by medical advice), and, having gained more or less of the vices it leads to, improve rapidly from having their minds occupied at regular hours, in the same way as healthy people of their age. The second principle of which I will speak now is, that we must, in the interest of our nervous patients, much more than in our own, give the confidence and hope in the treatment we recommend. In hysterical and all nervous complaints allied with it, and also in hypochondria, and in several other neuroses, a great hope of cure will do much to work out the cure. No doubt you will say, How to give hope? I answer that the best means for that purpose is to have hope ourselves, and to express our hope with the accent of conviction. And as you would ask, How can we command hope in ourselves? I answer that the very knowledge of the truth of the principle I am now speaking of is enough to render one hopeful. I need not repeat that I am now speaking only of those neuroses in which the power of the mind upon the body is immense, and so much so that in some forms of these neuroses a sudden or almost sudden cure is not very rare.

IV. *Special modes of treatment in periodical nervous affections.*

—It is not my intention to speak now of the well known useful influence of sulphate of quinine against perfectly periodical attacks of neuralgia, of epilepsy, etc.; I only wish to speak of a method of perturbation of the nervous system which I have employed with great advantage in some of those cases of local convulsions, of attacks of epilepsy, of hysteria, etc., which either recur nearly at fixed periods, or are preceded by a warning that gives time to make use of the means I will now mention. In a case of spasm of some muscles of the jaw and face, preceded by a sensation of pricking in the cheek, and occurring several times a-day, in a boy seven years old, I found that violent exercise on a swing always prevented the fit when the patient had time to run to the swing before the muscular contraction had begun. By that means, which never failed, the boy was many hundred times saved from his attack before being completely cured of it, which occurred on the coming out of a molar tooth, which, however, had not given the least pain. The beneficial influence of this mode of perturbation of the nervous system I have observed since in a number of cases of hysteria and epilepsy. A great many other means of changing the state of the nervous system have been employed with some benefit by other physicians or by myself. Among these means I will merely mention here the following:—A ligature round one or several limbs (even when there is no evident aura) tied strongly and suddenly; a pretty sharp pinching of the skin; a rather large dose of an emetic taken with a great quantity of water; a cold shower-bath on the back; the application of an interrupted and powerful electro-magnetic current; a dose of twelve, fifteen, or eighteen grains of sulphate of quinine about an hour before the expected attack; an enema of a drastic medicine; the inhalation of chloroform, etc. I will give more details on these important perturbing means, in the lectures on hysteria, neuralgia, and epilepsy.

